# Intra-articular injections for knee osteoarthritis management: Analysis of cost-effectiveness

**DOI:** 10.1016/j.ocarto.2025.100641

**Published:** 2025-06-10

**Authors:** Hanna Mass, Jamie E. Collins, Catherine Yang, David J. Hunter, Morgan H. Jones, Love Tsai, Stephen P. Messier, Tuhina Neogi, Jeffrey N. Katz, Elena Losina

**Affiliations:** a Orthopedic and Arthritis Center for Outcomes Research, Department of Orthopedic Surgery, Brigham and Women's Hospital, Boston, MA, USA; b Harvard Medical School, Boston, MA, USA; c Rheumatology Department, Royal North Shore Hospital and Sydney Musculoskeletal Health, Kolling Institute, University of Sydney, Sydney, New South Wales, Australia; d J.B. Snow Biomechanics Laboratory, Department of Health and Exercise Science, Wake Forest University, Winston-Salem, NC, USA; e Section of Rheumatology, Boston University Chobanian & Avedisian School of Medicine, Boston, MA, USA; f Policy and Innovation eValuation in Orthopaedic Treatments (PIVOT) Center, Department of Orthopaedic Surgery, Brigham and Women's Hospital, Boston, MA, USA

## Abstract

**Objective:**

Intra-articular injections (IAI) are commonly used to treat knee pain in persons with knee osteoarthritis (OA). We sought to determine the value of commonly used IAIs in knee OA management.

**Methods:**

We used the validated Osteoarthritis Policy Model (OAPol) to assess the value of saline, corticosteroid (CS), hyaluronic acid (HA), and platelet-rich plasma (PRP) IAIs in knee OA management. We conducted a meta-analysis of high quality studies to estimate IAI-specific pain reduction. We assumed that repeat CS injections increase the risk of OA progression threefold in the base case. We determined the value of specific IAIs with incremental cost-effectiveness ratios (ICERs). We conducted sensitivity analyses to account for uncertainty in input parameters.

**Results:**

In the base case, ICERs were $8300/QALY for saline compared to no injection, $54,500/QALY for HA compared to saline, and $112,100/QALY for PRP compared to HA. CS was dominated (more costly, less effective) by saline. **If saline was not included, ICER for HA was reduced to $22,400/QALY.** In sensitivity analyses that assumed CS does not increase OA progression, ICERs were $6000/QALY for CS compared to no injection, HA dominated compared to CS. ICER for PRP was estimated at $151,300/QALY. ICERs for PRP were higher than currently accepted willingness to pay thresholds. PRP ICER ranges were most sensitive to discontinuation probability and cost.

**Conclusions:**

CS could offer good value for knee OA management if the impact on OA progression is small. **Value of PRP depends greatly on its price, with current prices leading to value exceeding well-accepted cost-effectiveness thresholds**. Better data on the impact of CS on OA progression and pain efficacy related to PRP would offer critical insights for policymakers into the value of specific IAIs in the management of knee OA.

## 1 Introduction

Knee osteoarthritis (OA) is a common and disabling condition affecting more than 360 million people worldwide [1]. The US accounts for around 8.3 ​% of these cases, with approximately 30 million affected persons [2]. This burden will likely increase as the population ages [3]. Intra-articular injections (IAIs) are commonly utilized to treat pain and delay total knee replacement (TKR) in patients with knee OA who do not receive adequate relief from nonsteroidal anti-inflammatory drugs (NSAIDs), diet and exercise, and/or physical therapy (PT) [4].

Different classes of IAIs are associated with varying levels of pain relief, duration of relief, impact on OA progression, and cost [4,5]. CS IAIs provide short-term pain relief and are relatively inexpensive [6]. In a survey of American Association of Hip and Knee Surgeon members published in 2021, all respondents (N ​= ​537) noted using corticosteroid (CS) injections in their practice [7]. Concern about continued CS usage has emerged in light of evidence suggesting that the IAI may accelerate OA progression, although other studies have not supported this association [[8], [9], [10]]. Over the past decade, the healthcare market has also experienced an influx of orthobiologic injection treatments such as hyaluronic acids (HA) and platelet-rich plasma (PRP) [11,12]. HA and PRP are more expensive alternatives to CS. They may provide longer-term pain relief, although the quality of studies reporting longer durations of pain relief varies greatly. Uncertainty about the efficacy of these injections is reflected in OA treatment guidelines, many of which recommend against their use [[13], [14], [15]]. **Despite the lack of uniformity for IAI recommendations, their use in day-to-day clinical practice continues to grow, likely due to limited options for pain management in patients with knee OA and low concerns regarding IAI safety.**

**Evidence of efficacy is not a sole cause of implementing a specific regimen into clinical practice. As we all live in the era of limited resources, maximizing the benefits within limited health care budgets is one of the key objectives of any health care system. Cost-effectiveness analysis is an established research design.** Given the extensive usage and conflicting evidence of IAI efficacy, it is important to identify the circumstances under which the choice of one IAI over another might be clinically and economically justified. Since PRP injections are not currently FDA-approved and patients pay out-of-pocket, unnecessary PRP prescription carries important implications for patients in the US. Previous IAI cost-effectiveness analyses were limited by comparing only two IAIs and not generally extending beyond a single course of treatment [16]. Our objective is to estimate the cost-effectiveness of three, multi-course IAI treatment regimens (CS, HA, and PRP) alongside IAI-sparing and saline placebo scenarios.

## 2 Methods

### 2.1 Analytic overview

We used the Osteoarthritis Policy (OAPol) Model to evaluate the cost-effectiveness of five different IAI strategies embedded into sequential, usual care regimens for knee OA. Our primary outcome was the incremental cost-effectiveness ratio (ICER), calculated as the ratio of the difference in lifetime medical costs and the difference in quality-adjusted life expectancy between two consecutive strategies. Strategies were consecutively ranked in order of increasing costs and could be cost-effective, dominated, or weakly dominated. Strategies were considered *cost-effective* if they offered more QALYs and fewer costs than the competing strategy, *dominated* if a competing strategy had lower costs and more QALYs, and *weakly dominated* if a competing strategy had higher costs but a lower ICER. The strategy offering the most QALYs from among those whose incremental cost-effectiveness ratios (ICERs) are below a prespecified willingness-to-pay (WTP) threshold was considered the *most cost-effective* strategy. Within the US context, WTP thresholds typically fall between $50,000 and $150,000.

We modeled healthcare costs in 2023 USD and discounted costs and benefits at 3 ​% annually [17]. We conducted analyses from both a healthcare perspective and a societal perspective. The healthcare perspective incorporates all direct medical costs associated with the strategy to the healthcare system and the patient. The societal perspective captures all direct and indirect costs associated with the OA and its management, including lost productivity due to knee OA and non-medical costs associated with opioid pain relief [18]. We averaged lifetime costs and QALEs over five runs of five million subjects for each strategy to obtain stable estimates. We evaluated the robustness of our conclusions in the face of input parameter uncertainty by conducting deterministic and probabilistic sensitivity analyses (PSA).

### 2.2 Model structure

The OAPol Model is a widely published state-transition Monte Carlo microsimulation of knee OA natural disease and treatment [19,20]. The model follows subjects who transition through various health states based on prespecified probabilities. Health states are defined by clinical characteristics such as OA structural severity (Kellgren-Lawrence (KL) grade) and pain levels, comorbidities (cardiovascular disease, diabetes, cancer, chronic obstructive pulmonary disease), and BMI-based obesity class. Each health state is associated with a mortality risk governed by age/sex/race and comorbidities, as informed by US lifetables. Health states are also associated with a preference-based QoL utility (with 0 corresponding to death and 1 to perfect health), as well as OA- and non-OA-related medical costs that accumulate until death. QoL utilities are determined by an individual's number of comorbidities, obesity status, pain level, and age.

Pain is derived from the Western Ontario and McMaster Universities Osteoarthritis (WOMAC) pain scale (0–100, 100 worst) [21]. OA treatment regimens relieve pain by prespecified decrements, defined by treatment efficacy and starting pain level. Those who receive pain relief initially may lose the benefit; such “pain failure” is defined in a probabilistic manner. Treatment regimens also accumulate costs and carry a risk of adverse events. Subjects may discontinue a regimen due to reaching the prespecified maximum time on the regimen, experiencing serious adverse events or pain failure, or wishing to withdraw for miscellaneous reasons. Discontinuation from a regimen triggers evaluation for eligibility for the next regimen in the treatment sequence. In addition to regimen discontinuation, the model utilizes the concept of efficacy sustainability, where subjects continue experiencing the benefits of a regimen even when they are no longer receiving treatment.

### 2.3 Treatment strategies under consideration

We followed subjects from treatment initiation until death. All subjects were assumed to have persistent pain despite a round of NSAIDs and PT. We modeled five initial treatment strategies: 1) IAI-sparing, 2) saline (placebo), 3) CS, 4) HA, and 5) PRP. The saline and CS regimens consisted of a single injection repeated every 3 months [6,7]. The HA injection regimen consisted of a series of 1–3 injections repeated every 6 months [22]. The PRP injection regimen consisted of a series of 1–3 injections repeated every 12 months [23]. Subjects remained on IAIs for a maximum of five years, until they experienced “pain failure,” or discontinued for other reasons. If subjects experienced pain failure after the first injection series, they continued to receive a second series. However, if pain failure was experienced in any subsequent injection series, subjects discontinued the injection regimen and moved on to the next regimen. All strategies were followed by the potential use of opioids, TKR, and revision TKR. Eligibility for each regimen depended on OA severity.

#### 2.3.1 Input parameters

A summary of key inputs is presented in Table 1.

**Table 1:** Select OAPol Model inputs.

Simulated Cohort Demographics		
Parameter	Mean (SD) or percent	Reference
Age	61.2 (8.6)	See Supplemental Table 1
Sex		[24]
Female	55 ​%	
Male	45 ​%	
BMI, kg/m^2^	30.6 (6.8)	[25]
Pain, WOMAC	50.8 (16.4)	See Supplemental Table 1

Injection pain relief		

Starting WOMAC pain score	Mean (SD) change in WOMAC	Reference

**CS**		See Supplemental Table 1
15–40	14.79 (7.40)	
41–70	27.13 (13.60)	
71+	41.99 (21.00)	
**HA**		See Supplemental Table 1
15–40	12.92 (6.46)	
41–70	25.26 (12.63)	
71+	37.12 (18.56)	
**PRP**		See Supplemental Table 1
15–40	19.62 (3.81)	
41–70	24.96 (9.98)	
71+	34.82 (17.41)	
**Saline**		See Supplemental Table 1
15–40	5.79 (2.90)	
41–70	17.13 (8.57)	
71+	30.49 (15.24)	
Injection cost		

Injection type	Estimate	Reference

CS	$337	[34]
HA	$851	[34]
PRP	$2767	[28]
Saline	$331	[34]

Injection toxicity characteristics		

Parameter	Estimate	Reference

**Minor toxicity (skin flare)**		
Annual probability	24.0 ​%	[44]
Quality of life decrement	0.998	[45]
Cost	$0.00	[47]
**Major toxicity (sepsis)**		
Annual probability	0.0013 ​%	[47]
Quality of life decrement	0.976	[46]
Cost	$14,535	[47]

### 2.4 Cohort characteristics

We modeled a cohort that was 55 ​% female with a mean age of 61.2 (8.6) and a mean BMI of 30.6 ​kg/m^2^ (6.8). 71 ​% of simulated subjects were white non-Hispanic, 15.9 ​% were white Hispanic, and 13 ​% were African American non-Hispanic. All individuals entered the simulation with knee OA, with an even distribution between KL grades 2 and 3 and a mean pain of 50.8 (0) on a 0–100 scale (100 worse). We derived mean age and baseline pain from published trials (Technical Appendix, Supplemental Table 1). We derived sex and race distribution from US Census data [24], and BMI from the National Health and Nutrition Examination Survey [25].

### 2.5 Treatment characteristics: injection regimen efficacy

In the model, treatment efficacy is modeled as the mean decrement in pain on the WOMAC scale following treatment initiation, stratified by starting pain level. To determine the efficacy of various injection regimens, we performed a detailed literature review (Technical Appendix, Section 1.1). We included RCTs that reported mean pain scores at the various time points post-randomization. For CS, we required pain scores at three months. For HA, we required scores reported at three and six months. For PRP, we required scores reported at three and twelve months. To standardize across injection regimen doses, we included only RCTs that used a single injection for CS and series of 1–3 injections for HA and PRP. In addition, we used only medium/high molecular weight HA formulations to minimize inconsistencies within the HA injection regimen. To assess the methodological quality of all identified RCTs, one reviewer (HM) assigned each study a Jadad score [26]. We set a cut-off score of four or higher to ensure that only double-blinded RCTs with sound methodological quality were included. Additional details are in Supplemental Table 1 in the Technical Appendix. We included 12 RCTs for CS, 24 for HA, and 8 for PRP IAIs. Of these RCTs, 11 contained a saline comparator arm used to inform the saline regimen.

We standardized data for treatment efficacy to a 0–100 pain scale and used a random-effects meta-analysis to estimate IAI-specific pain reductions. We validated these OAPol model inputs by comparing the simulated pain decrements from the OAPol model with the random effects estimates from the meta-analysis for each injection type at specific time points: 3 months for CS and saline, 6 months for HA, and 12 months for PRP. These time points were determined based on literature reporting the estimated efficacy duration for each injection [6,[27], [28], [29]]. We include further information on derivations in Supplemental Tables 2 and 4, and on validation in Supplemental Fig. 1.

### 2.6 Treatment characteristics: injection regimen discontinuation and sustainability

Subjects remained on a regimen until they experienced a loss of treatment efficacy (i.e., “pain failure” described above) or discontinued for other reasons. We modeled different probabilities of discontinuation across the four injections (Supplemental Table 3) [[30], [31], [32]]. Additional details are provided in the Technical Appendix, Section 1.2. For subjects who did not experience a pain failure or discontinue the IAI regimen, we assumed they could receive the IAI for up to five years. Between 1 ​% and 17 ​% of subjects remain on an IAI regimen for five years (Table 3).

We assumed sustainability probability to vary between 5 ​% and 20 ​%, and time spent sustaining to vary between six and 24 months. Additional details on sustainability are in the Technical Appendix (Section 1.2, Supplemental Table 3).

### 2.7 Treatment characteristics: cost

We used the Health Care Common Procedure Coding System (HCPCS), a standardized coding system produced by the Centers for Medicare and Medicaid Services, to determine the cost of medical procedures, supplies, and products [33]. The cost for each IAI series included costs of an initial office visit (HCPCS 99213), IAI administration (HCPCS 20610), and lidocaine injection (HCPCS J2001), derived from the Medicare Physician Fee Schedule [34]. We derived the cost of saline, CS, and HA medication from the Medicare Drug Fee schedule and estimated the cost of PRP from the literature [28]. We assumed that subjects completed an entire series of injections and were billed accordingly. The total cost for administration of one complete series of saline, CS, HA, and PRP IAI, inflated to 2023 USD, was $331, $337, $851, and $2767 respectively.

The base case costs are reported from both the healthcare and societal perspective. In the societal perspective, we included indirect costs such as loss of wages due to OA pain or surgery and all costs from the healthcare perspective [35]. Patients may also incur indirect costs due to opioid use.

### 2.8 Treatment characteristics: adverse events

Recent studies have raised concern regarding the risk of accelerated OA progression and cartilage damage with continued use of CS injections over time [8,9]. To account for this potential adverse effect, we conducted a base case analysis assuming that repeat CS injections increase the risk of OA progression threefold [8]. Other studies, however, have suggested that CS does not increase the risk of OA progression [10,36]. In sensitivity analyses, we varied the impact of CS injections on OA progression with risk ratios between 1 and 5.

Due to a lack of published data on the adverse events of PRP and HA injections, we assumed that adverse events other than the increased risk of OA progression occurred at a rate of 24 ​% per year, were the same across all IAIs, and carried a marginal QoL decrement primarily attributable to site irritation and infection.

### 2.9 Treatment characteristics: opioids and TKR

Upon entering the model, subjects are assumed to have failed an initial course of NSAIDs and PT. All IAI strategies were followed by UC, which consists of the possible use of opioids, TKR, and revision TKR [35]. To model opioid use, we calibrated treatment offer and acceptance so that 5 ​% of subjects used opioids.

## 3 Sensitivity analysis

### 3.1 Deterministic sensitivity analysis

We performed one-way sensitivity analyses in which we varied the following parameters: PRP cost ($1000 to $5000), general discontinuation (±5 ​%,±10 ​% from the base case value), PRP discontinuation (±10 ​%,±20 ​% from the base case value), duration of IAI efficacy sustainability (zero to five times the base case duration), risk of accelerated OA progression for CS regimen (RR ​= ​1–5), starting pain (30–60), and starting KL grade (100 ​% KL2 to 100 ​% KL3). The large range for PRP cost is intended to account for the substantial variability in the published literature: one study of top orthopaedic hospitals in the United States found that the price for a single PRP injection could range from 350 to 2815 dollars, meaning that one series of 1–3 injections could cost between 1359 and 8754 dollars [37]. The ranges for the other parameters reflect either the levels of uncertainty found in the literature or discretionary choices due to a lack of evidence.

We also modeled two additional scenarios: one where TKR was not offered and one where all subjects received only one IAI series. In addition, we performed a two-way sensitivity analysis where we varied the two parameters with the widest ICER ranges from the one-way sensitivity analysis.

### 3.2 Probabilistic sensitivity analysis

We examined the impact of uncertainty in parameters shown to be influential in the one-way sensitivity analysis by varying them simultaneously in PSA. For HA, we varied pain efficacy, percentage of subjects sustaining pain benefits, duration of sustainability, and discontinuation. We varied the same parameters for PRP with the addition of cost. We varied the rate of OA progression for the CS intervention. PSA parameters and their respective distributions can be found in the Technical Appendix, Supplemental Table 5. We depicted the results of the PSA in a cost-effectiveness acceptability curve, illustrating the percent of the times each IAI strategy had an ICER below the prespecified WTP, considering a range of WTP thresholds from $0 to $250,000 $/QALY.

## 4 Results

### 4.1 Base case analysis

Results of the base case analysis from the healthcare perspective are shown in Table 2. The estimated ICERs were $8300/QALY for saline compared to no injection, $54,500/QALY for HA compared to saline, and $112,100/QALY for PRP compared to HA. CS was dominated by saline, indicating that CS provided fewer QALYs for more cost compared to saline. If CS does not increase the risk of OA progression, saline is dominated, CS has an ICER of $6000/QALY compared to no injection, HA is dominated (lower QALY, higher cost) compared to CS, and PRP has an ICER of $151,300/QALY compared to CS (Table 2). The magnitude of the increased risk of OA progression due to repeat CS use led to substantial variability in CS ICERs (Supplemental Table 8). ICERs for CS ranged from $6000 to $39,000 as the relative risk of progression increased from 1 to 1.5 and was dominated at relative risks >1.5 (Technical Appendix Table 8).

**Table 2:** Costs, clinical benefits, and incremental cost-effectiveness ratios for select intra-articular injections without indirect costs/loss of productivity.

Strategy	COST	QALYs	ICER (incremental)	ICER, excluding saline (incremental)
*Base Case: CS increases risk of OA progression*				
**No injection**	$154,600	9.87		
**Saline**	$154,900	9.91	$8300	–
**CS**	$155,300	9.88	D	d
**HA**	$155,900	9.93	$54,500	$22,400
**PRP**	$159,800	9.96	$112,100	$112,100
*Selected Sensitivity Analysis: CS does not increase risk of OA progression*				
**No injection**	$154,600	9.87		
**Saline**	$154,900	9.91	d	–
**CS**	$154,900	9.93	$6000	$6000
**HA**	$155,900	9.93	D	D
**PRP**	$159,800	9.96	$151,300	$151,300

CS = Corticosteroid, HA = Hyaluronic Acid, PRP = Platelet-Rich Plasma, QALY ​= ​Quality Adjusted Life Year, ICER = Incremental Cost-Effectiveness Ratio. ICERs were rounded to the nearest 100.

D ​= ​Dominated, costs more, has lower benefit (QALY).

d ​= ​extended dominance, ICER is greater than the ICER for the next strategy. Cost and ICERs are rounded to $100s.

**Table 3:** Percentage of simulated subjects on each injection series, stratified by type of injection.

	Injection Series 1	Injection Series 2	Injection Series 3	Injection Series 4	Injection Series 6	Injection Series 9
**Saline**	100 ​%	94 ​%	39 ​%	21 ​%	7 ​%	1 ​%
**CS**	100 ​%	92 ​%	49 ​%	32 ​%	15 ​%	4 ​%
**HA**	100 ​%	85 ​%	34 ​%	19 ​%	6 ​%	–
**PRP**	100 ​%	80 ​%	31 ​%	17 ​%	–	–

In the base case analysis conducted from the societal perspective, saline had an ICER of $4,500, CS was dominated by HA, HA had an ICER of $51,400/QALY compared to saline, and PRP had an ICER of $111,900 compared to HA (Supplemental Table 6). When saline was removed as a comparator, CS had a higher ICER than HA and was weakly dominated (indicating that CS cost more per QALY than HA), whereas HA had an ICER of $22,400/QALY (Table 2).

### 4.2 Deterministic sensitivity analysis

**Fig. 1:**
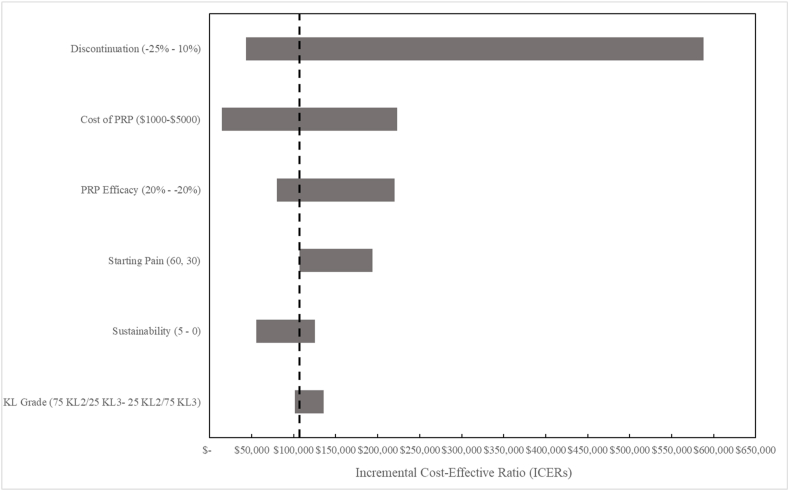
**One-way sensitivity analysis for PRP.** We performed one-way sensitivity analyses varying discontinuation, cost of PRP, PRP efficacy, starting pain, sustainability, and KL grade. This tornado diagram is a simultaneous display of all these analyses. The dashed line represents the base case ICER for PRP. Each horizontal bar represents the range of ICERS that results as each parameter is varied across its plausible range while holding all other parameters at their base-case values. Wider ranges and longer bars illustrate which parameters exert greater influence on a treatment's ICER. By convention, we display the bars from the longest (denoting the parameter generating the widest uncertainty) at the top to the shortest (denoting the parameter generating the least uncertainty) at the bottom.

To present select results of one-way sensitivity analyses on PRP, we use a tornado diagram (Fig. 1). Tornado diagrams utilize bars to show the range of ICERs that result from varying the parameter, with longer bars representing greater sensitivity to parameter assumptions and therefore lower relative robustness. PRP is most sensitive to discontinuation probability: ICERs for PRP relative to HA increase from $112,100 (base case) to $587,900 when discontinuation probability is increased by 10 ​% (Fig. 1). PRP is also sensitive to cost: ICERs are $14,300, $112,100 (base case), and $223,300 for costs of $1,000, $2767 (base case), and $5,000, respectively. The ICER for PRP becomes larger than a WTP threshold of $100,000 ​at a cost between $2000 and $2767. Although PRP is not as sensitive to variation in sustainability of effect as it is to discontinuation or cost, sustainability duration nonetheless carries important implications for the value of PRP. Increasing sustainability from the base case twofold decreases the ICER from $112,100 (base case) to $88,800; increasing sustainability fivefold decreases the ICER to $55,400.

Within our one-way sensitivity analyses, we also investigated no TKR scenarios. When TKR was not offered, saline IAI had an ICER of $18,700 compared to no injection, HA had an ICER of $44,300 compared to saline, and PRP had an ICER of $83,700 compared to HA.

**Fig. 2:**
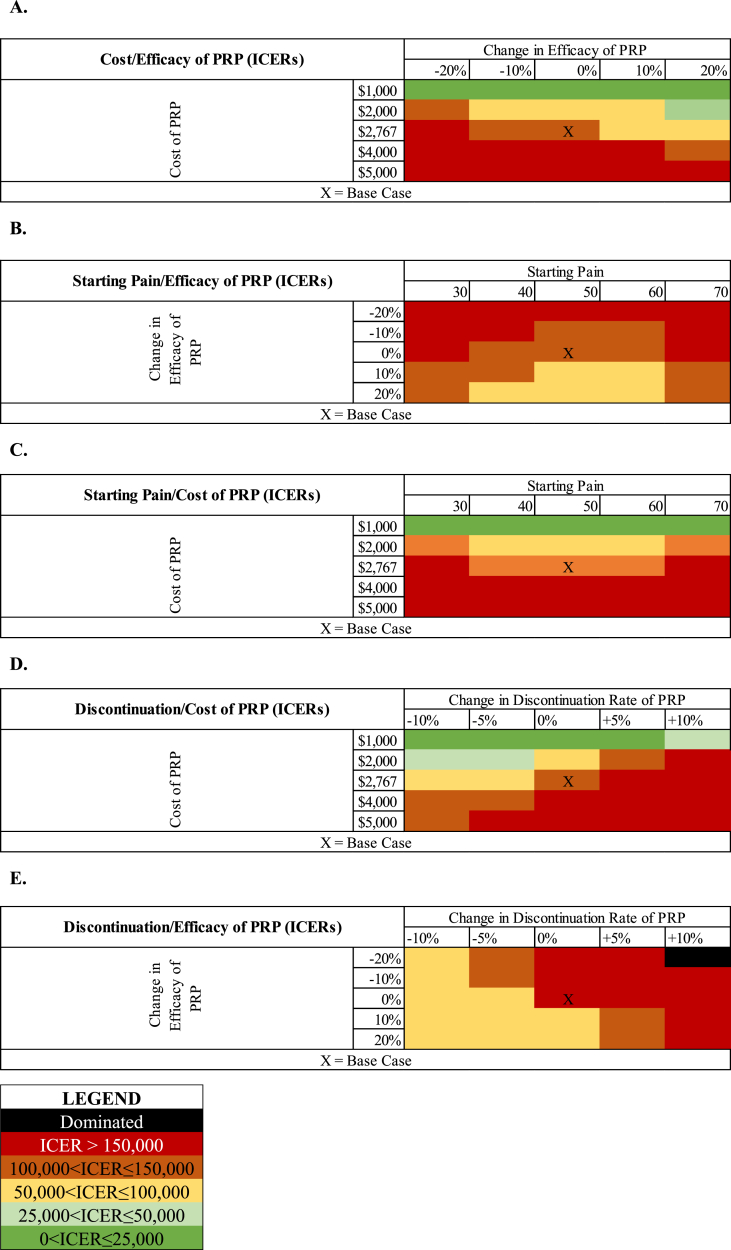
**Results of two-way sensitivity analysis for PRP.** We performed two-way sensitivity analyses varying two parameters at a time, with all other parameters held at base-case values. The resulting heat maps show how the ICER is affected by varying each set of parameters. Fig. 2A illustrates ICERs corresponding to the simultaneous variation of PRP cost and efficacy. Fig. 2B illustrates ICERs corresponding to the simultaneous variation of starting pain and PRP efficacy. Fig. 2C illustrates ICERs corresponding to the simultaneous variation of starting pain and PRP cost. Fig. 2D illustrates ICERs corresponding to the simultaneous variation of PRP discontinuation probability and cost. Fig. 2E illustrates ICERs corresponding to the simultaneous variation of PRP discontinuation probability and PRP efficacy.

Fig. 2 shows the results of our two-way sensitivity analyses of PRP parameters, where we simultaneously varied cost and efficacy, efficacy and starting pain, starting pain and cost, discontinuation and cost, and discontinuation and efficacy. When varying cost and efficacy, low PRP costs always result ICERs under $25,000 (Fig. 2a). As the cost increases, the efficacy of PRP must increase to maintain cost-effectiveness. When varying efficacy and starting pain, PRP efficacy 20 ​% worse than the base case scenario always resulted in ICERs greater than $150,000 (Fig. 2b). As PRP efficacy increases, we see a pain-dependent effect where both lower pain and higher pain make the IAI not cost-effective. A similar phenomenon occurs when simultaneously varying starting pain and cost: as PRP price increases from $1,000, both lower and higher pain increase PRP's ICER relative to the base case (Fig. 2c). This pattern is interrupted when PRP cost becomes greater than $4000. The scenarios varying discontinuation with cost and discontinuation with efficacy follow more linear relationships (Fig. 2c and d).

### 4.3 Probabilistic sensitivity analysis

**Fig. 3:**
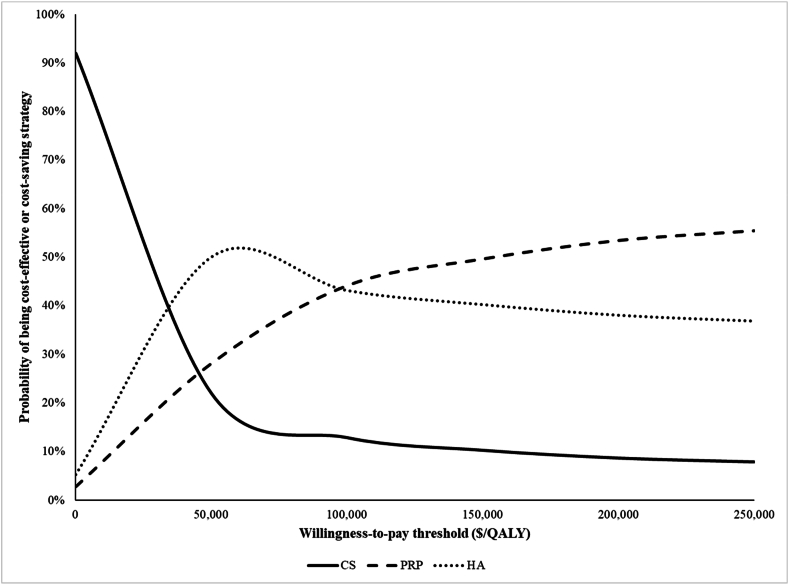
**Cost-effectiveness acceptability curve.** We conducted a probabilistic sensitivity analysis of the CS, PRP, and HA strategies. For both PRP and HA, we varied pain efficacy, percentage of subjects sustaining pain benefits after discontinuing treatment, duration of sustainability, and discontinuation probability. For PRP alone, we also varied cost. For CS, we only varied the rate of OA progression. ICERS were calculated by comparing the CS, PRP, and HA strategies. The probability of being a cost-effective or cost-saving strategy is plotted at WTP thresholds from $0 to $250,000. CS is represented by the solid line, PRP by the dashed line, and HA by the dotted line.

We varied key input parameters simultaneously in PSA, including pain efficacy, discontinuation, and accelerated rate of OA progression for CS (Supplemental Table 5). Fig. 3 depicts the percentage of scenarios (out of 1000) in which CS, HA, and PRP were cost-effective at a given WTP threshold. CS was cost-effective at WTP thresholds of $50,000 and $100,000 per QALY in 22 ​% and 13 ​% of scenarios, respectively. At the same thresholds, HA was cost-effective in 50 ​% and 43 ​% of scenarios, respectively. Lastly, PRP was cost-effective in 28 ​% and 44 ​% of scenarios at the same thresholds. None of the IAI-based strategies were cost-effective at levels >60 ​% for any WTP<$200,000/QALY.

## 5 Discussion

Knee OA is a prevalent, costly, and incurable disease. In recent years, IAIs have been increasingly used in clinical practice to treat OA pain, despite uncertainty surrounding their efficacy. We conducted cost-effectiveness analyses examining the value of several intraarticular injection strategies in the management of symptomatic knee OA. The results indicate that, at a WTP threshold of $100,000, both saline and HA are cost-effective. Our results reflect findings in the literature that saline placebo has been shown to contribute to pain relief and functional improvement in knee OA [38].

The value of CS greatly depends on the relative risk of OA progression. CS ICERs are well below WTP thresholds when there is no associated increased risk of OA progression, while CS ICERs are dominated by saline when the risk ratio is above 1.6. A recent meta-analysis reports doubled odds of progression associated with CS; in our sensitivity analysis, CS was dominated by HA when progression odds were doubled [39]. However, two recent studies suggest that there is no increased risk of progression associated with CS injection [10,36]. Determining whether CS confers a risk of progression should be a major research priority.

Cost-effectiveness analyses comparing a single course of CS or HA IAIs with acetaminophen have reported that under a $50,000 WTP threshold, both were shown to be cost-effective treatments [40,41]. In addition, HA products investigated in a single-payer economic evaluation were found to be cost-effective at a WTP threshold under $10,000 when compared to conventional care [16]. However, the authors mentioned sources of bias such as the reliance of industry funding in the included studies, and the analysis was conducted in a short timeframe over 6 months. None of these studies modeled an increased risk of OA progression associated with CS injection. As we have shown, the conclusions regarding the cost-effectiveness of CS or HA greatly depend on this risk of OA [16].

Studies of the value of PRP have yielded a range of findings. Raeissadat et al. performed a Monte Carlo simulation reporting that compared to plasma rich in growth factors, HA, and intra-articular injection of ozone, PRP was the dominant strategy with an ICER of $7600 [42]. Raeissadat et al. estimated medical costs according to tariffs from public medical centers in Iran, where HA is costly, while PRP remains comparatively cheaper as the preparation kits are manufactured in Iran. The estimated cost of PRP in their study was $90.5, compared to $2767 in our base case analysis [42]. Similarly, Samuelson et al. utilized a decision tree model and reported that, at one year, a series of PRP injections, compared to HA was more effective, with an ICER of $12,600 (2019 USD) [43]. The authors derived their health-related utility values from a systematic review including six studies (only one of which we used in our analysis) and also cited a substantially larger reduction in WOMAC pain for PRP compared to HA, relative to the findings from our systemic review [43]. The authors found initial WOMAC pain reductions of 23.86 and 8.65 for PRP and HA, respectively, compared to 16.96 and 22.26 in our meta-analysis. This highlights the sensitivity of the cost-effectiveness to efficacy, as shown in our deterministic sensitivity analyses.

One limitation of our work is the lack of standardization across data sources and between different injection regimens. There is a large variation in the formulations and doses of injections available for PRP and HA. By including all types of high molecular weight HA formulations and PRP preparations for 1–3 injection series, our results are generalizable across injection types, though the efficacy of specific formulations may vary beyond the scope of this analysis. To standardize treatment protocols, we assumed that all patients adhere to the entire injection series, which is another limitation. Stem cell injections provide an alternative treatment option for knee OA. However, we choose not to include them in our analysis due to the lack of high-quality published data.

This work highlights a major limitation in existing literature regarding the uncertainty around key input parameters. While we conducted a thorough literature review for information on efficacy, adverse effects, adherence, and discontinuation, much of the data were conflicting, as in the case of the increased risk of OA progression associated with CS, or lacking, as in the case of discontinuation rates. By including only studies with a Jadad score of 4+, we attempted to minimize bias. Due to this uncertainty, we varied parameters widely in our sensitivity analyses and found many of the results not robust to these variations. CS could be extremely cost-effective, or not cost-effective, depending on OA progression risk, while PRP was similarly sensitive to discontinuation probability. Establishing greater certainty around these parameters would allow for a more definitive evaluation of cost-effectiveness. **Quantifying uncertainty allows** **one** **to assess the benefits of obtaining more information regarding model inputs. Since no strategies were deemed cost-effective at WTP<$200,000** **in** **more than 60 % of replications, this** **points to a critical need of definitive,** **realistically-powered clinical studies establishing the clinical benefits of IAIs for patients with knee OA,** **particularly** **given** **the** **high utilization of these regimens.** A value-of-information analysis could shed light on which information would be most valuable to reduce uncertainty.

Nonetheless, our results reveal the potential value of various IAIs in knee OA treatment, with HA being the most cost-effective IAI option under a WTP threshold of <25,000 if CS does accelerate OA progression and saline is not considered as a viable treatment modality. **PRP is currently not FDA approved and not reimbursed by Medicare or other payers. Our analyses pointed that at current pricing, its cost** **exceeds most** **currently accepted willingness-to-pay thresholds.**

**Evidence of efficacy is not sufficient for adoption in day-to-day care. As we all live in the era of limited resources, maximizing the benefits within limited health care budgets is one of the key objectives of any health care system. In the paradigm of high use, despite low effect size and high cost (PRP, HA), formal evaluation of cost-effectiveness offers critical insights for payers and decision makers in either promoting or demoting the use of specific intra-articular injections.** Policymakers and physicians may consider these results when determining the cost-effectiveness of injection treatments and engaging in shared decision-making with patients regarding OA treatment.

## Author contributions

Substantial contributions to study conception and design – all authors.

Substantial contributions to the acquisition of data – HM, JC.

Substantial contributions to analysis and interpretation of data – all authors.

Drafting the article or revising it critically for important intellectual content – all authors.

Final approval of the version of the article to be published – all authors.

## Funding

Supported by National Institute of Arthritis and Musculoskeletal and Skin Diseases (NIAMS) grants R01 AR074290, P30 AR072571, and P30 AR072577.

## Declaration of competing interest

Outside of the listed NIH/NIAMS funding, all authors have not received any other financial support for this manuscript.

## References

[1] Kang Y., Liu C., Ji Y. (2024). The burden of knee osteoarthritis worldwide, regionally, and nationally from 1990 to 2019, along with an analysis of cross-national inequalities. Arch. Orthop. Trauma Surg..

[2] Safiri S., Kolahi A.A., Smith E. (2020). Global, regional and national burden of osteoarthritis 1990-2017: a systematic analysis of the Global Burden of Disease Study 2017. Ann. Rheum. Dis..

[3] GBD 2021 Osteoarthritis Collaborators (2023). Global, regional, and national burden of osteoarthritis, 1990-2020 and projections to 2050: a systematic analysis for the Global Burden of Disease Study 2021. Lancet Rheumatol.

[4] Testa G., Giardina S.M.C., Culmone A. (2021). Intra-articular injections in knee osteoarthritis: a review of literature. J. Funct. Morphol. Kinesiol.

[5] Ding J.B., Hu K. (2021). Injectable therapies for knee osteoarthritis. Reumatologia.

[6] Saltychev M., Mattie R., McCormick Z., Laimi K. (2020). The magnitude and duration of the effect of intra-articular corticosteroid injections on pain severity in knee osteoarthritis: a systematic review and meta-analysis. Am. J. Phys. Med. Rehabil..

[7] Blankstein M., Lentine B., Nelms N.J. (2021). Common practices in intra-articular corticosteroid injection for the treatment of knee osteoarthritis: a survey of the American association of hip and knee surgeons membership. J. Arthroplast..

[8] Zeng C., Lane N.E., Hunter D.J. (2019). Intra-articular corticosteroids and the risk of knee osteoarthritis progression: results from the Osteoarthritis Initiative. Osteoarthr. Cartil..

[9] McAlindon T.E., LaValley M.P., Harvey W.F. (2017). Effect of intra-articular triamcinolone vs saline on knee cartilage volume and pain in patients with knee osteoarthritis: a randomized clinical trial. JAMA.

[10] Bucci J., Chen X., LaValley M. (2022). Progression of knee osteoarthritis with use of intraarticular glucocorticoids versus hyaluronic acid. Arthritis Rheumatol..

[11] Lo G.H., LaValley M., McAlindon T., Felson D.T. (2003). Intra-articular hyaluronic acid in treatment of knee osteoarthritis: a meta-analysis. JAMA.

[12] Pretorius J., Habash M., Ghobrial B., Alnajjar R., Ellanti P. (2023). Current status and advancements in platelet-rich plasma therapy. Cureus.

[13] Brophy R.H., Fillingham Y.A. (2022). AAOS clinical practice guideline summary: management of osteoarthritis of the knee (nonarthroplasty), third edition. J. Am. Acad. Orthop. Surg..

[14] Bannuru R.R., Osani M.C., Vaysbrot E.E. (2019). OARSI guidelines for the non-surgical management of knee, hip, and polyarticular osteoarthritis. Osteoarthr. Cartil..

[15] Kolasinski S.L., Neogi T., Hochberg M.C. (2020). American College of Rheumatology/Arthritis Foundation guideline for the management of osteoarthritis of the hand, hip, and knee. Arthritis Care Res..

[16] Rosen J., Sancheti P., Fierlinger A., Niazi F., Johal H., Bedi A. (2016). Cost-effectiveness of different forms of intra-articular injections for the treatment of osteoarthritis of the knee. Adv. Ther..

[17] Sanders G.D., Neumann P.J., Basu A. (2016). Recommendations for conduct, methodological practices, and reporting of cost-effectiveness analyses: second panel on cost-effectiveness in health and medicine. JAMA.

[18] Kim D.D., Silver M.C., Kunst N., Cohen J.T., Ollendorf D.A., Neumann P.J. (2020). Perspective and costing in cost-effectiveness analysis, 1974-2018. Pharmacoeconomics.

[19] Bensen G.P., Rogers A.C., Leifer V.P. (2023). Does gabapentin provide benefit for patients with knee OA? A benefit-harm and cost-effectiveness analysis. Osteoarthr. Cartil..

[20] Losina E., Smith K.C., Paltiel A.D. (2019). Cost-effectiveness of diet and exercise for overweight and obese patients with knee osteoarthritis. Arthritis Care Res..

[21] McConnell S., Kolopack P., Davis A.M. (2001). The Western Ontario and McMaster Universities osteoarthritis Index (WOMAC): a review of its utility and measurement properties. Arthritis Care Res..

[22] Altman R., Hackel J., Niazi F., Shaw P., Nicholls M. (2018). Efficacy and safety of repeated courses of hyaluronic acid injections for knee osteoarthritis: a systematic review. Semin. Arthritis Rheum..

[23] Bansal H., Leon J., Pont J.L. (2021). Platelet-rich plasma (PRP) in osteoarthritis (OA) knee: correct dose critical for long term clinical efficacy. Sci. Rep..

[24] (2023). Population and housing unit estimates Tables. https://www.census.gov/programs-surveys/popest/data/tables.html.

[25] (2018). National health and nutrition examination survey. https://www.cdc.gov/nchs/nhanes/index.htm.

[26] Jadad A.R., Moore R.A., Carroll D. (1996). Assessing the quality of reports of randomized clinical trials: is blinding necessary?. Control. Clin. Trials.

[27] Chavda S., Rabbani S.A., Wadhwa T. (2022). Role and effectiveness of intra-articular injection of hyaluronic acid in the treatment of knee osteoarthritis: a systematic review. Cureus.

[28] Piuzzi N.S., Ng M., Kantor A. (2019). What is the price and claimed efficacy of platelet-rich plasma injections for the treatment of knee osteoarthritis in the United States?. J. Knee Surg..

[29] Filardo G., Previtali D., Napoli F., Candrian C., Zaffagnini S., Grassi A. (2021). PRP injections for the treatment of knee osteoarthritis: a meta-analysis of randomized controlled trials. Cartilage.

[30] Maricar N., Parkes M.J., Callaghan M.J. (2017). Structural predictors of response to intra-articular steroid injection in symptomatic knee osteoarthritis. Arthritis Res. Ther..

[31] Liu S.H., Dubé C.E., Driban J.B., McAlindon T.E., Eaton C.B., Lapane K.L. (2017). Patterns of intra-articular injection use after initiation of treatment in patients with knee osteoarthritis: data from the osteoarthritis initiative. Osteoarthr. Cartil..

[32] Navarro-Sarabia F., Coronel P., Collantes E. (2011). A 40-month multicentre, randomised placebo-controlled study to assess the efficacy and carry-over effect of repeated intra-articular injections of hyaluronic acid in knee osteoarthritis: the AMELIA project. Ann. Rheum. Dis..

[33] Healthcare common procedure coding system (HCPCS) Accessed 5/February/2023.

[34] Medicare physician fee schedule. https://www.cms.gov/medicare/physician-fee-schedule/search.

[35] Losina E., Paltiel A.D., Weinstein A.M. (2015). Lifetime medical costs of knee osteoarthritis management in the United States: impact of extending indications for total knee arthroplasty. Arthritis Care Res..

[36] Latourte A., Rat A.C., Omorou A. (2022). Do glucocorticoid injections increase the risk of knee osteoarthritis progression over 5 Years?. Arthritis Rheumatol..

[37] Tiao J., Wang K., Herrera M. (2024). There is wide variation in platelet-rich plasma injection pricing: a United States nationwide study of top orthopaedic hospitals. Clin. Orthop. Relat. Res..

[38] Altman R.D., Devji T., Bhandari M., Fierlinger A., Niazi F., Christensen R. (2016). Clinical benefit of intra-articular saline as a comparator in clinical trials of knee osteoarthritis treatments: a systematic review and meta-analysis of randomized trials. Semin. Arthritis Rheum..

[39] Ibad H.A., Kasaeian A., Ghotbi E. (2023). Longitudinal MRI-defined cartilage loss and radiographic joint space narrowing following intra-articular corticosteroid injection for knee osteoarthritis: a systematic review and meta-analysis. Osteoarthr. Imag.

[40] Bannuru Jbw R.R., Kent D.M., Schmid C.H., McAlindon T.E. (2015). Intra-articular corticosteroids may be a cost-effective streategy for short-term management of knee osteoarthritis. Osteoarthr. Cartil..

[41] Migliore A., Integlia D., Pompilio G., Di Giuseppe F., Aru C., Brown T. (2019). Cost-effectiveness and budget impact analysis of viscosupplementation with hylan G-F 20 for knee and hip osteoarthritis. Clin. Outcomes Res..

[42] Raeissadat S.A., Rahimi M., Rayegani S.M., Moradi N. (2023). Cost-utility analysis and net monetary benefit of Platelet Rich Plasma (PRP), intra-articular injections in compared to Plasma Rich in Growth Factors (PRGF), Hyaluronic Acid (HA) and ozone in knee osteoarthritis in Iran. BMC Muscoskelet. Disord..

[43] Samuelson E.M., Ebel J.A., Reynolds S.B., Arnold R.M., Brown D.E. (2020). The cost-effectiveness of platelet-rich plasma compared with hyaluronic acid injections for the treatment of knee osteoarthritis. Arthroscopy.

[44] Ayral X. (2001). Injections in the treatment of osteoarthritis. Best Practice & Research Clinical Rheumatology.

[45] Yen Z.S., Lai M.S., Wang C.T., Chen L.S., Chen S.C., Chen W.J., Hou S.M. (2004). Cost-effectiveness of treatment strategies for osteoarthritis of the knee in Taiwan. J Rheumatology.

[46] Talmor D., Greenberg D., Howell M.D., Lisbon A., Novack V., Shapiro N. (2008 Apr). The costs and cost-effectiveness of an integrated sepsis treatment protocol. Crit Care Med.

[47] HCUP National Inpatient Sample (NIS) (2012). Healthcare Cost and Utilization Project (HCUP).

