# Supplementary material for: Intra-articular injections for knee osteoarthritis management: Analysis of cost-effectiveness
Source: Osteoarthr Cartil Open. 2025 Jun 10;7(3):100641. doi: 10.1016/j.ocarto.2025.100641 (PMC12284352; doi:10.1016/j.ocarto.2025.100641)
Supplement: Multimedia component 1 [file mmc1.docx]

**Alternative Intra-Articular Injections for Knee Osteoarthritis Management: Analysis of Cost-effectiveness**

Table Of Contents

[1. Model Inputs 2](#_Toc183434320)

[1.1 Pain Efficacy: Meta-Analysis 2](#_Toc183434321)

[1.2 Discontinuation and Sustainability 3](#_Toc183434322)

[2. Model Validations 4](#_Toc183434323)

[3. Probabilistic Sensitivity Analysis Inputs 6](#_Toc183434324)

[4. Additional Cost-Effectiveness Analysis Results 8](#_Toc183434325)

[6. References 11](#_Toc183434326)

# Model Inputs

## Pain Efficacy: Meta-Analysis

We used a random-effects meta-analysis to derive treatment efficacy model inputs. First, we performed an individual search on PubMed for CS, HA, and PRP injections published between 2010 and 2021 using the keywords: “X injection AND knee osteoarthritis.” We also reviewed citations from a published literature review on relevant injections to ensure all relevant papers were included.^1^ After obtaining a preliminary list of papers, we excluded studies that were either not RCTs or small RCTs (sample size < 30). Additional exclusion criteria specific to each injection type are detailed in the table below. Data extracted from each study are shown in Supplemental Table 2.

| **HA Injections** |  |
| --- | --- |
| Initial Pub med download: | 274 |
| Published after 2010 | 190 |
| Relevant to HA and knee OA* | 102 |
| Reports mean pain score | 91 |
| JADAD Score 4+ | 48 |
| Sample size 30+ | 36 |
| Reports 3- & 6-month time points° | 27 |
| Series 1-3 injections | 23 |
| M/H Molecular weight | 22 |
| Additional papers from citation searching | 2 |
| Total Papers Included | **24^2-25^** |

Supplemental Table 1: Literature review for various injections.

| **Corticosteroid Injections:** |  |
| --- | --- |
| Initial Pub med download: | 97 |
| Published after 2010 | 81 |
| Relevant to CS and knee OA* | 36 |
| Reports mean pain score | 30 |
| JADAD Score 4+ | 14 |
| Sample size 30+ | 10 |
| Reports 3-month time point° | 10 |
| Single injection series | 10 |
| Additional papers from citation searching | 2 |
| Total Papers Included | **12^6,7,19,26-34^** |

| **PRP Injections** |  |
| --- | --- |
| Initial Pub med download: | 91 |
| Published after 2010 | 91 |
| Relevant to PRP and knee OA* | 49 |
| Reports mean pain score | 46 |
| JADAD Score 4+ | 21 |
| Sample size 30+ | 12 |
| Reports 3- & 12-month time points° | 7 |
| Series 1-3 injections | 7 |
| Additional papers from citation searching | 1 |
| Total Papers Included | **8^3,21,25,30,35-38^** |

*Excluded papers that were not relevant, not RCTs, included an injection other than the relevant injection, included an intervention such as surgery or PT, and/or not in English.

°Time points include plus/minus four weeks.

Supplemental Table 2: Pain data included in meta-analysis.

## 1.2 Discontinuation and Sustainability

Due to a lack of research on these parameters, we assumed many probabilities for discontinuation, pain sustainability, and time sustaining through internal discussions with clinicians (Supplemental Table 3).

Discontinuation probabilities were informed by a 2017 study by Maricar et al., reporting that 73.4% of patients injected with CS were OMERACT-OARSI “responders” at short-term follow-up and saw favorable results.^39^ In terms of general trends, discontinuation is slightly higher for HA and PRP relative to CS/saline (placebo) due to cost, side effects, and/or increased uncertainty regarding efficacy. Discontinuation probability varied by injection series, with subsequent series having lower probabilities of discontinuation than the initial injection series.

Sustainability probability is assumed the same across all IAIs. Additionally, for all injection types, we modeled two assumptions for time spent sustaining and averaged the results. One assumption accounted for subjects in whom the injection relieved an initial pain flare which allowed them to resume PT or exercise. These subjects would therefore experience longer pain sustainability. The other assumption accounted for subjects who experienced some pain relief but were not able to sustain this relief for as long as the first group. The time sustaining after the first injection is the same across all IAIs but is slightly longer in HA and PRP regimens for subsequent injections due to data on increased response to HA injections where 80.5% of participants qualified as OMERACT-OARSI responders at 40 months.^40^ We varied the duration of sustainability in sensitivity analyses to account for the uncertainty in these assumptions.

| Supplemental Table 3: Injection discontinuation and sustainability model inputs. | | | | |
| --- | --- | --- | --- | --- |
|  | | **Discontinuation** | **% Pain Sustainability** | **Time Sustaining** |
| **CS/Saline** | Inj. 1 | 10% | 7.5% | 6 months |
|  |  |  | 7.5% | 24 months |
|  | Inj. 2 | 30% | 15% | 6 months |
|  |  |  | 5% | 9 months |
|  | Inj. 3+ | 15% | 20% | 6 months |
|  |  |  | 5% | 9 months |
| **HA** | Inj. 1 | 20% | 7.5% | 6 months |
|  |  |  | 7.5% | 24 months |
|  | Inj. 2 | 45% | 15% | 9 months |
|  |  |  | 5% | 15 months |
|  | Inj. 3+ | 25% | 20% | 9 months |
|  |  |  | 5% | 12 months |
| **PRP** | Inj. 1 | 25% | 7.5% | 6 months |
|  |  |  | 7.5% | 24 months |
|  | Inj. 2 | 50% | 15% | 9 months |
|  |  |  | 5% | 15 months |
|  | Inj. 3+ | 30% | 20% | 9 months |
|  |  |  | 5% | 12 months |

# Model Validations

We validated treatment efficacy by comparing the literature efficacy with the model output and calibrating WOMAC pain decrements experienced by simulated subjects with WOMAC pain decrements derived from the random-effects model.

| Supplemental Table 4: Injection pain efficacy validation. Efficacy values represent the mean decrement in pain on the WOMAC scale. | | |
| --- | --- | --- |
| **Timepoint (Months)** | **Literature Efficacy** | **Model Output** |
| *Corticosteroid Injection (CS)* | | |
| 3 | 22.13 | 22.90 |
| *Platelet Rich Plasma (PRP)* | | |
| 3 | 18.59 | 18.26 |
| 12 | 16.96 | 17.67 |
| *Saline* | | |
| 3 | 14.13 | 13.70 |
| *Hyaluronic Acid (HA)* | | |
| 3 | 15.93 | 15.85 |
| 6 | 22.26 | 22.40 |

Supplemental Figure 1: Pain trajectories for various injections. The average pain of simulated subjects was plotted over time. The black line represents the pain trajectory for subjects who did not receive injections, the yellow line represents saline, the solid orange line represents CS with OA progression, the dotted orange line represents CS without OA progression, the red line represents PRP, and the brown line represents HA injections.

# Probabilistic Sensitivity Analysis Inputs

Supplemental Table 5 lists the parameters tested in probabilistic sensitivity analyses, wherein all selected parameters were varied simultaneously and drawn from a probability distribution. We conducted 1000 simulations each for the CS, HA, and PRP strategies.

| **Supplemental Table 5. Probabilistic Sensitivity Analysis Distributions** | | | | | | |
| --- | --- | --- | --- | --- | --- | --- |
| **PRP Intervention** | **Variable** | **Base Case**  **(Mean)** | **SD** | **Distribution Type** | **Alpha** | **Beta** |
|  | Duration of Treatment Efficacy | Series 1: 5 Years | N/A | Poisson | Series 1: Lambda = 5 Years | N/A |
|  |  | Series 2: 4 Years |  |  | Series 2: Lambda =  4 Years |  |
|  |  | Series 3+:  3.5 Years |  |  | Series 3+: Lambda =  3.5 Years |  |
|  | Discontinuation | Series 1: 25% | Series 1:  13% | Beta | Series 1:  0.6875 | Series 1:  2.0625 |
|  |  | Series 2:  50% | Series 2:  25% |  | Series 2:  0.375 | Series 2:  0.375 |
|  |  | Series 3+:  30% | Series 3+:  15% |  | Series 3+:  0.625 | Series 3+:  1.458 |
|  | Cost of PRP | $2767 | $1,384 | Gamma | 4 | 646.5 |
|  | Proportion of Subjects Experiencing Sustainability | Series 1: 7.5% | N/A | Beta Binomial | Series 1: 7.5 | Series 1: 92.5 |
|  |  | Series 2:  10% |  |  | Series 2:  10 | Series 2:  90 |
|  |  | Series 3: 12.5% |  |  | Series 3: 12.5 | Series 3: 87.5 |
|  | Pain Efficacy (Decrement in WOMAC Pain) | Pain Group 3: 19.62 | Pain Group 3: 9.81 | Normal | N/A | N/A |
|  |  | Pain Group 4: 24.96 | Pain Group 4: 12.48 |  |  |  |
| **HA Intervention** | Duration of Treatment Efficacy | Series 1: 5 Years | N/A | Poisson | Series 1: Lambda = 5 Years | N/A |
|  |  | Series 2: 4 Years |  |  | Series 2: Lambda = 4 Years |  |
|  |  | Series 3+:  3.5 Years |  |  | Series 3+: Lambda = 3.5 Years |  |
|  | Discontinuation | Series 1: 20% | Series 1:  10% | Beta | Series 1:  0.75 | Series 1:  3 |
|  |  | Series 2:  45% | Series 2:  23% |  | Series 2:  0.4375 | Series 2:  0.5347 |
|  |  | Series 3+:  25% | Series 3+:  13% |  | Series 3+:  0.6875 | Series 3+:  2.0625 |
|  | Proportion of Subjects Experiencing Sustainability | Series 1: 7.5% | N/A | Beta Binomial | Series 1: 7.5 | Series 1: 92.5 |
|  |  | Series 2:  10% |  |  | Series 2:  10 | Series 2:  90 |
|  |  | Series 3: 12.5% |  |  | Series 3: 12.5 | Series 3: 87.5 |
|  | Pain Efficacy (Decrement in WOMAC Pain) | Pain Group 3: 12.92 | Pain Group 3: 6.46 | Normal | N/A | N/A |
|  |  | Pain Group 4: 25.26 | Pain Group 4: 12.63 |  |  |  |
| **CS Intervention** | Relative Risk of KL Progression | 3.02 | 1.51 | Log Normal | N/A | N/A |

# Additional Cost-Effectiveness Analysis Results

We evaluated the base case from both the healthcare and the societal perspective. The societal perspective includes indirect costs associated with a treatment strategy, such as loss of work productivity. The results of this analysis are presented in Supplemental Table 6.

| Supplemental Table 6. Costs, clinical benefits, and incremental cost-effectiveness ratios for select intra-articular injections including indirect costs/loss of productivity. | | | |
| --- | --- | --- | --- |
| **Strategy** | **COST** | **QALYs** | **ICER** |
| *CS increases risk of OA progression* | | | |
| No Injection | $ 157,100 | 9.87 |  |
| Saline | $ 157,300 | 9.91 | $ 4,500 |
| CS (OA) | $ 157,800 | 9.88 | D |
| HA | $ 158,300 | 9.93 | $ 51,400 |
| PRP | $ 162,200 | 9.96 | $ 111,900 |
| CS = Corticosteroid, HA = Hyaluronic Acid, PRP = Platelet Rich Plasma, QALY = Quality Adjusted Life Year, ICER = Incremental Cost-Effectiveness Ratio. ICERs were rounded to the nearest 100.  D = Dominated: costs more, has lower benefit (i.e., QALYs) | | | |

To reflect real-world scenarios in which saline is not a viable option for most patients, we present analyses with saline removed as a comparator in Supplemental Table 7.

| Supplemental Table 7. Costs, clinical benefits, and incremental cost-effectiveness ratios for select intra-articular injections without indirect costs/loss of productivity with saline removed. | | | |
| --- | --- | --- | --- |
| **Strategy** | **COST** | **QALYs** | **ICER** |
| *CS increases risk of OA progression* | | | |
| No Injection | $154,600 | 9.87 |  |
| CS | $155,300 | 9.88 | d |
| HA | $155,900 | 9.93 | $22,400 |
| PRP | $159,800 | 9.96 | $112,100 |
| CS = Corticosteroid, HA = Hyaluronic Acid, PRP = Platelet Rich Plasma, QALY = Quality Adjusted Life Year, ICER = Incremental Cost-Effectiveness Ratio. ICERs were rounded to the nearest 100.  D = Dominated, costs more, has lower benefit (QALY)  d = Weekly dominated, ICER is greater than the ICER for the next strategy | | | |

CS is most cost-effective at willingness-to-pay thresholds under $150,000 when we assume that CS does not impact OA progression. To test the impact of accelerated OA progression and cost-effectiveness, we ran one-way deterministic analyses in which we varied the hazard ratio of OA progression between one and five in increments of 0.1. The results (see Supplemental Table 8) demonstrate that at hazard ratio 1.6, CS becomes weakly dominated. At hazard ratios 1.7 and above, CS becomes dominated.

| **Supplemental Table 8.** Table showing positive, linear relationship between the hazard ratio of OA acceleration and CS intra-articular injection ICERs. | |
| --- | --- |
| **Hazard Ratio of OA Acceleration** | **CS ICER (compared to Saline or No Injection)** |
| **1** | $6,000 |
| **1.1** | $7,200 |
| **1.2** | $7,900 |
| **1.3** | $12,500 |
| **1.4** | $22,000 |
| **1.5** | $39,000 |
| **1.6** | Weakly Dominated* |
| **1.7 – 5** | Dominated** |
| *ICER ($/QALY) is greater than the ICER for the next strategy  **Costs more and has lower benefit (QALY) | |

# 6. References

1. Anil U, Markus DH, Hurley ET, et al. The efficacy of intra-articular injections in the treatment of knee osteoarthritis: A network meta-analysis of randomized controlled trials. *Knee.* 2021;32:173-182.

2. Park YB, Kim JH, Ha CW, Lee DH. Clinical Efficacy of Platelet-Rich Plasma Injection and Its Association With Growth Factors in the Treatment of Mild to Moderate Knee Osteoarthritis: A Randomized Double-Blind Controlled Clinical Trial As Compared With Hyaluronic Acid. *Am J Sports Med.* 2021;49(2):487-496.

3. Cole BJ, Karas V, Hussey K, Pilz K, Fortier LA. Hyaluronic Acid Versus Platelet-Rich Plasma: A Prospective, Double-Blind Randomized Controlled Trial Comparing Clinical Outcomes and Effects on Intra-articular Biology for the Treatment of Knee Osteoarthritis. *Am J Sports Med.* 2017;45(2):339-346.

4. Conrozier T, Eymard F, Afif N, Balblanc JC, Legré-Boyer V, Chevalier X. Safety and efficacy of intra-articular injections of a combination of hyaluronic acid and mannitol (HAnOX-M) in patients with symptomatic knee osteoarthritis: Results of a double-blind, controlled, multicenter, randomized trial. *Knee.* 2016;23(5):842-848.

5. Guo Y, Yang P, Liu L. Origin and Efficacy of Hyaluronan Injections in Knee Osteoarthritis: Randomized, Double-Blind Trial. *Med Sci Monit.* 2018;24:4728-4737.

6. Leighton R, Akermark C, Therrien R, et al. NASHA hyaluronic acid vs. methylprednisolone for knee osteoarthritis: a prospective, multi-centre, randomized, non-inferiority trial. *Osteoarthritis Cartilage.* 2014;22(1):17-25.

7. Tammachote N, Kanitnate S, Yakumpor T, Panichkul P. Intra-Articular, Single-Shot Hylan G-F 20 Hyaluronic Acid Injection Compared with Corticosteroid in Knee Osteoarthritis: A Double-Blind, Randomized Controlled Trial. *J Bone Joint Surg Am.* 2016;98(11):885-892.

8. Wang YC, Lee CL, Chen YJ, et al. Comparing the Efficacy of Intra-Articular Single Platelet-Rich Plasma(PRP) versus Novel Crosslinked Hyaluronic Acid for Early-Stage Knee Osteoarthritis: A Prospective, Double-Blind, Randomized Controlled Trial. *Medicina (Kaunas).* 2022;58(8).

9. van der Weegen W, Wullems JA, Bos E, Noten H, van Drumpt RA. No difference between intra-articular injection of hyaluronic acid and placebo for mild to moderate knee osteoarthritis: a randomized, controlled, double-blind trial. *J Arthroplasty.* 2015;30(5):754-757.

10. Bettonville M, Léon M, Margaux J, et al. Safety and efficacy of a single intra-articular injection of a novel enhanced protein solution (JTA-004) compared to hylan G-F 20 in symptomatic knee osteoarthritis: a randomized, double-blind, controlled phase II/III study. *BMC Musculoskelet Disord.* 2021;22(1):888.

11. Petrella RJ, Emans PJ, Alleyne J, Dellaert F, Gill DP, Maroney M. Safety and performance of Hydros and Hydros-TA for knee osteoarthritis: a prospective, multicenter, randomized, double-blind feasibility trial. *BMC Musculoskelet Disord.* 2015;16:57.

12. Sun SF, Hsu CW, Lin HS, Liou IH, Chen YH, Hung CL. Comparison of Single Intra-Articular Injection of Novel Hyaluronan (HYA-JOINT Plus) with Synvisc-One for Knee Osteoarthritis: A Randomized, Controlled, Double-Blind Trial of Efficacy and Safety. *J Bone Joint Surg Am.* 2017;99(6):462-471.

13. Hangody L, Szody R, Lukasik P, et al. Intraarticular Injection of a Cross-Linked Sodium Hyaluronate Combined with Triamcinolone Hexacetonide (Cingal) to Provide Symptomatic Relief of Osteoarthritis of the Knee: A Randomized, Double-Blind, Placebo-Controlled Multicenter Clinical Trial. *Cartilage.* 2018;9(3):276-283.

14. Pavelka K, Uebelhart D. Efficacy evaluation of highly purified intra-articular hyaluronic acid (Sinovial(®)) vs hylan G-F20 (Synvisc(®)) in the treatment of symptomatic knee osteoarthritis. A double-blind, controlled, randomized, parallel-group non-inferiority study. *Osteoarthritis Cartilage.* 2011;19(11):1294-1300.

15. Takamura J, Seo T, Strand V. A Single Intra-Articular Injection of Gel-200 for Treatment of Symptomatic Osteoarthritis of the Knee Is More Effective than Phosphate Buffered Saline at 6 Months: A Subgroup Analysis of a Multicenter, Randomized Controlled Trial. *Cartilage.* 2019;10(4):417-422.

16. Berenbaum F, Grifka J, Cazzaniga S, et al. A randomised, double-blind, controlled trial comparing two intra-articular hyaluronic acid preparations differing by their molecular weight in symptomatic knee osteoarthritis. *Ann Rheum Dis.* 2012;71(9):1454-1460.

17. Maheu E, Avouac B, Dreiser RL, Bardin T. A single intra-articular injection of 2.0% non-chemically modified sodium hyaluronate vs 0.8% hylan G-F 20 in the treatment of symptomatic knee osteoarthritis: A 6-month, multicenter, randomized, controlled non-inferiority trial. *PloS one.* 2019;14(12):e0226007.

18. Maheu E, Zaim M, Appelboom T, et al. Comparative efficacy and safety of two different molecular weight (MW) hyaluronans F60027 and Hylan G-F20 in symptomatic osteoarthritis of the knee (KOA). Results of a non inferiority, prospective, randomized, controlled trial. *Clin Exp Rheumatol.* 2011;29(3):527-535.

19. Housman L, Arden N, Schnitzer TJ, et al. Intra-articular hylastan versus steroid for knee osteoarthritis. *Knee Surg Sports Traumatol Arthrosc.* 2014;22(7):1684-1692.

20. Cortet B, Lombion S, Naissant B, Vidovic E, Bruyère O. Non-Inferiority of a Single Injection of Sodium Hyaluronate Plus Sorbitol to Hylan G-F20: A 6-Month Randomized Controlled Trial. *Adv Ther.* 2021;38(5):2271-2283.

21. Filardo G, Di Matteo B, Di Martino A, et al. Platelet-Rich Plasma Intra-articular Knee Injections Show No Superiority Versus Viscosupplementation: A Randomized Controlled Trial. *Am J Sports Med.* 2015;43(7):1575-1582.

22. Giarratana LS, Marelli BM, Crapanzano C, et al. A randomized double-blind clinical trial on the treatment of knee osteoarthritis: the efficacy of polynucleotides compared to standard hyaluronian viscosupplementation. *Knee.* 2014;21(3):661-668.

23. Chevalier X, Jerosch J, Goupille P, et al. Single, intra-articular treatment with 6 ml hylan G-F 20 in patients with symptomatic primary osteoarthritis of the knee: a randomised, multicentre, double-blind, placebo controlled trial. *Ann Rheum Dis.* 2010;69(1):113-119.

24. de Campos GC, Rezende MU, Pailo AF, Frucchi R, Camargo OP. Adding triamcinolone improves viscosupplementation: a randomized clinical trial. *Clin Orthop Relat Res.* 2013;471(2):613-620.

25. Bansal H, Leon J, Pont JL, et al. Platelet-rich plasma (PRP) in osteoarthritis (OA) knee: Correct dose critical for long term clinical efficacy. *Sci Rep.* 2021;11(1):3971.

26. McAlindon TE, LaValley MP, Harvey WF, et al. Effect of Intra-articular Triamcinolone vs Saline on Knee Cartilage Volume and Pain in Patients With Knee Osteoarthritis: A Randomized Clinical Trial. *JAMA.* 2017;317(19):1967-1975.

27. Conaghan PG, Hunter DJ, Cohen SB, et al. Effects of a Single Intra-Articular Injection of a Microsphere Formulation of Triamcinolone Acetonide on Knee Osteoarthritis Pain: A Double-Blinded, Randomized, Placebo-Controlled, Multinational Study. *J Bone Joint Surg Am.* 2018;100(8):666-677.

28. Mendes JG, Natour J, Nunes-Tamashiro JC, Toffolo SR, Rosenfeld A, Furtado RNV. Comparison between intra-articular Botulinum toxin type A, corticosteroid, and saline in knee osteoarthritis: a randomized controlled trial. *Clin Rehabil.* 2019;33(6):1015-1026.

29. Babaei-Ghazani A, Najarzadeh S, Mansoori K, et al. The effects of ultrasound-guided corticosteroid injection compared to oxygen-ozone (O(2)-O(3)) injection in patients with knee osteoarthritis: a randomized controlled trial. *Clin Rheumatol.* 2018;37(9):2517-2527.

30. Nunes-Tamashiro JC, Natour J, Ramuth FM, et al. Intra-articular injection with platelet-rich plasma compared to triamcinolone hexacetonide or saline solution in knee osteoarthritis: A double blinded randomized controlled trial with one year follow-up. *Clin Rehabil.* 2022;36(7):900-915.

31. Bodick N, Lufkin J, Willwerth C, et al. An intra-articular, extended-release formulation of triamcinolone acetonide prolongs and amplifies analgesic effect in patients with osteoarthritis of the knee: a randomized clinical trial. *J Bone Joint Surg Am.* 2015;97(11):877-888.

32. Lomonte AB, de Morais MG, de Carvalho LO, Zerbini CA. Efficacy of Triamcinolone Hexacetonide versus Methylprednisolone Acetate Intraarticular Injections in Knee Osteoarthritis: A Randomized, Double-blinded, 24-week Study. *J Rheumatol.* 2015;42(9):1677-1684.

33. Joshi Jubert N, Rodríguez L, Reverté-Vinaixa MM, Navarro A. Platelet-Rich Plasma Injections for Advanced Knee Osteoarthritis: A Prospective, Randomized, Double-Blinded Clinical Trial. *Orthop J Sports Med.* 2017;5(2):2325967116689386.

34. Askari A, Gholami T, NaghiZadeh MM, Farjam M, Kouhpayeh SA, Shahabfard Z. Hyaluronic acid compared with corticosteroid injections for the treatment of osteoarthritis of the knee: a randomized control trail. *SpringerPlus.* 2016;5:442.

35. Bennell KL, Paterson KL, Metcalf BR, et al. Effect of Intra-articular Platelet-Rich Plasma vs Placebo Injection on Pain and Medial Tibial Cartilage Volume in Patients With Knee Osteoarthritis: The RESTORE Randomized Clinical Trial. *JAMA.* 2021;326(20):2021-2030.

36. Di Martino A, Boffa A, Andriolo L, et al. Leukocyte-Rich versus Leukocyte-Poor Platelet-Rich Plasma for the Treatment of Knee Osteoarthritis: A Double-Blind Randomized Trial. *Am J Sports Med.* 2022;50(3):609-617.

37. Yurtbay A, Say F, Çinka H, Ersoy A. Multiple platelet-rich plasma injections are superior to single PRP injections or saline in osteoarthritis of the knee: the 2-year results of a randomized, double-blind, placebo-controlled clinical trial. *Arch Orthop Trauma Surg.* 2022;142(10):2755-2768.

38. Chu J, Duan W, Yu Z, et al. Intra-articular injections of platelet-rich plasma decrease pain and improve functional outcomes than sham saline in patients with knee osteoarthritis. *Knee Surg Sports Traumatol Arthrosc.* 2022;30(12):4063-4071.

39. Maricar N, Parkes MJ, Callaghan MJ, et al. Structural predictors of response to intra-articular steroid injection in symptomatic knee osteoarthritis. *Arthritis Res Ther.* 2017;19(1):88.

40. Navarro-Sarabia F, Coronel P, Collantes E, et al. A 40-month multicentre, randomised placebo-controlled study to assess the efficacy and carry-over effect of repeated intra-articular injections of hyaluronic acid in knee osteoarthritis: the AMELIA project. *Ann Rheum Dis.* 2011;70(11):1957-1962.
